# An SSR-SNP Linkage Map of the Parasitic Weed *Orobanche cumana* Wallr. Including a Gene for Plant Pigmentation

**DOI:** 10.3389/fpls.2019.00797

**Published:** 2019-06-19

**Authors:** Álvaro Calderón-González, Nicolas Pouilly, Stéphane Muños, Xavier Grand, Marie Coque, Leonardo Velasco, Begoña Pérez-Vich

**Affiliations:** ^1^Instituto de Agricultura Sostenible (IAS) – Consejo Superior de Investigaciones Científicas (CSIC), Córdoba, Spain; ^2^Laboratoire des Interactions Plantes Micro-organismes (LIPM), UMR CNRS-INRA 2594-441, Castanet-Tolosan, France; ^3^Biogemma SAS, Paris, France

**Keywords:** linkage map, plant pigmentation, segregating populations, sunflower, sunflower broomrape

## Abstract

Sunflower broomrape (*Orobanche cumana* Wallr.) is a holoparasitic plant that causes major yield losses to sunflower crops in the Old World. Efforts to understand how this parasitic weed recognizes and interacts with sunflowers are important for developing long-term genetic resistance strategies. However, such studies are hampered by the lack of genetic tools for *O. cumana*. The objectives of this research were to construct a genetic linkage map of this species using SSR and SNP markers, and mapping the *P_g_* locus that is involved in plant pigmentation. The genetic map was developed from the progenies of a cross between the *O. cumana* inbred lines EK-12 and EK-A1, which originated from populations belonging to two distant and geographically separated gene pools identified in Spain. The inbred lines also differed in plant pigmentation, with EK-A1 lacking anthocyanin pigmentation (*p_g_p_g_* genotype). A genetic map comprising 26 SSR and 701 SNP markers was constructed, which displayed 19 linkage groups (LGs), corresponding to the 19 chromosome pairs of *O. cumana*. The total length of the map was 1795.7 cM, with an average distance between two adjacent positions of 2.5 cM and a maximum map distance of 41.9 cM. The *P_g_* locus mapped to LG19 between the SNP markers OS02468 and OS01653 at 7.5 and 3.4 cM, respectively. This study constitutes the first linkage map and trait mapping study in *Orobanche* spp., laying a key foundation for further genome characterization and providing a basis for mapping additional traits such as those having a key role in parasitism.

## Introduction

Sunflower broomrape (*Orobanche cumana* Wallr.) is a holoparasitic plant found in the wild from south-eastern Europe to central Asia parasitizing a few species of the Asteraceae, mainly *Artemisia* spp. As a parasitic weed, it parasitizes on sunflower and represents one of the most serious production constraints for this crop in many sunflower-producing countries, particularly in Central and Eastern Europe, Spain, Turkey, Israel, Iran, Kazakhstan, and China (Fernández-Martínez et al., 2015). Moreover, the parasite has spread in recent years to new countries where it had not been reported before such as France (Jestin et al., 2014), Tunisia (Amri et al., 2012), and Morocco (Nabloussi et al., 2018), and in countries where the parasite had been traditionally observed in specific areas, it continues spreading to new regions, such as the North of Spain (Fernández-Escobar et al., 2009; Malek et al., 2017).

The genetic interaction between broomrape and sunflower is in most cases governed by the gene-for-gene model for plant-pathogen interactions, in which resistance reactions are governed by the interaction of host genes for resistance and the corresponding pathogen genes for avirulence (Rodríguez-Ojeda et al., 2013b). This kind of interaction has led to the development of resistant sunflower cultivars based on vertical resistance mechanisms, and determines the occurrence of physiological races of broomrape that are controlled by these resistant genes in sunflower (Fernández-Martínez et al., 2015). Vranceanu et al. (1980) described five broomrape races in the early 80 s named as A to E and developed a set of sunflower differential lines to identify them, each carrying a single dominant gene (*Or1* through *Or5*, respectively) conferring resistance to the corresponding race. New races overcoming *Or5* resistance appeared from the middle 1990s onward in several countries such as Spain, Romania, Turkey, Bulgaria, Ukraine, and Russia (Fernández-Martínez et al., 2015). Initially, all of them were named as race F though the relationship between the different F races has not been studied. Nowadays, populations overcoming resistance sources to race F, named as races G and H, have been identified in most of these countries (Kaya et al., 2009; Pacureanu-Joita et al., 2009; Shindrova and Penchev, 2012; Antonova et al., 2013; Martín-Sanz et al., 2016). As mentioned for race F, no comparative studies have been conducted between races G and H reported in different countries. For races F and G, monogenic and dominant resistance in sunflower has also been reported (Pacureanu-Joita et al., 2004; Velasco et al., 2012).

As shown by broomrape race evolution, sunflower vertical resistance mechanisms are readily overcome by the parasite. For the development of long-term breeding strategies, it is essential to understand the genetic bases of the host-parasite interaction. However, this is currently hampered by the limited availability of genetic tools in *O. cumana*, since most of the research has been carried out on the crop host, the sunflower. The few genetic studies in *O. cumana* have mainly focused on population structure and genetic diversity analyses (Castejón-Muñoz et al., 1991; Gagne et al., 1998; Pineda-Martos et al., 2013, 2014; Guchetl et al., 2014; Molinero-Ruiz et al., 2014; Martín-Sanz et al., 2016). These studies concluded that the populations parasitizing sunflowers were characterized by low intra-population diversity and, in general, low differentiation between populations. Additionally, different mechanisms were postulated for explaining race evolution, such as single-gene mutations within local populations (Pineda-Martos et al., 2013), or genetic recombination of avirulence genes (Martín-Sanz et al., 2016). Classical genetic analyses in *O. cumana* were started just a few years ago. This in part might be due to the fact that working on genetics of holoparasitic plants signifies additional difficulties to obtain the plant material and segregating generations required for such studies, in such a way that all the plants-segregating generations have to be obtained by artificial inoculation on the corresponding host, which is a labor intensive and time-consuming procedure. Initially, Rodríguez-Ojeda et al. (2010) carried out basic studies for determining the feasibility of the use of inbreeding and hybridisation techniques for carrying out these genetic studies in *O. cumana*. Later, these authors isolated a line named EK-A1 from a natural mutant lacking anthocyanin pigmentation identified in a population of *O. cumana* from central Spain, and determined the inheritance of the trait evaluating the phenotype of segregating populations from crosses with plants of a normally pigmented line (Rodríguez-Ojeda et al., 2011). The authors concluded that the trait was controlled by partially dominant alleles at a single locus, which was named *P_g_*. Therefore, this trait showing a monogenic inheritance turned out to be an excellent candidate for conducting trait mapping studies in *O. cumana* genome. Additionally, the segregating population generated by Rodríguez-Ojeda et al. (2011) probed to be highly polymorphic, since it was constructed from parental lines belonging to the two genetically distant *O. cumana* gene pools that co-exist in Spain (the Gualdalquivir Valley and the Cuenca gene pools, in southern and central Spain, respectively, Pineda-Martos et al., 2013).

Genetic linkage maps are essential for studying the genome structure and organization. They are also valuable resources in parasitic weeds for locating genes that control traits of interest, such as avirulence or host specificity and ultimately might permit the positional cloning of these genes. In *Orobanche* spp., there have been no attempts to develop genetic linkage maps, and no trait mapping studies have been carried out to date. Genetic linkage maps in other parasitic weeds are also extremely limited. To our knowledge, the only study detailing a linkage map construction has been reported for *Striga hermonthica* (Del.) Benth., a parasitic weed of cereals, using amplified fragment length polymorphisms (AFLPs) (Pescott, 2013).

Since *O. cumana* genetic tools are essential to provide a better knowledge of the sunflower-*O. cumana* parasitic system for the development of knowledge-based control strategies, and these tools are so far extremely limited for this species and in general for *Orobanche* spp., the objectives of this research were to (i) develop a genetic linkage map in *O. cumana* using SSR and SNP markers, and (ii) map as a Mendelian trait the *Pg* locus involved in plant pigmentation, which was previously studied at the phenotypic level by Rodríguez-Ojeda et al. (2011).

## Materials and Methods

### Plant Material, Phenotyping and Mapping Population

**FIGURE 1:**
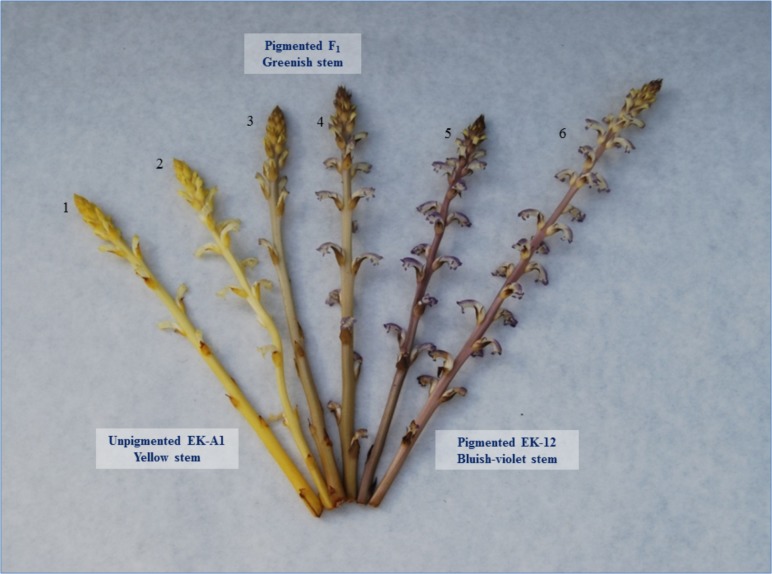
Unpigmented *O. cumana* EK-A1 plants (1, 2), partially pigmented heterozygotes (3, 4), and fully pigmented EK-12 plants (5, 6) showing yellow, greenish, and bluish-violet stems, respectively.

The mapping population consisted of 91 F_2_ plants and their corresponding F_2:3_ families derived from the cross between the *O. cumana* lines EK-A1 and EK-12, previously reported by Rodríguez-Ojeda et al. (2011). The EK-A1 plants lack pigmentation, having a yellow stem and a white corolla, whereas the EK-12 plants have normal pigmentation showing a bluish-violet stem and bluish to pale-violet corolla (Figure 1). This trait is controlled by partially dominant alleles at a single locus, referred to as *P_g_*. This was demonstrated by Rodríguez-Ojeda et al. (2011) through the independent evaluation of sixteen F_2_ populations (F_2_ plant generation) segregating for the plant pigmentation trait and the progenies of 120 F_2_ plants (F_3_ plant generation) evaluated in different years. All plants and generations were grown on the sunflower susceptible cultivar DMM as reported by Rodríguez-Ojeda et al. (2011). The growth conditions and phenotypic characterization for plant pigmentation of F_2_ and F_3_ plants were previously described by Rodríguez-Ojeda et al. (2011). For the molecular study, a F_2_ population consisting in 91 F_2_ plants for which (i) F_2:3_ families were available and (ii) there was sufficient F_3_ tissue, was used. Stem and corolla colors were visually evaluated at each 91 F_2_ plants and their corresponding F_2:3_ families (F_3_ plants), considering the phenotypes of the parental lines (unpigmented-yellow stem in EK-A1 plants and a fully pigmented-bluish-violet stem in EK-12 plants, Figure 1), and of the F_1_-heterozygous plants (greenish-intermediate pigmented, Figure 1; Rodríguez-Ojeda et al., 2011). Plant stems were scored as unpigmented (yellow) and pigmented (bluish violet-fully pigmented and greenish-intermediate pigmented). Pigmented plants (bluish violet and greenish) were included in the same class because of the large number of broomrape plants in some pots which made it difficult to distinguish greenish from bluish-violet plants. A Chi-square test was used to evaluate the proposed segregation ratio for the population used in the molecular study.

In order to evaluate the potential of the SNPs reported in this study for their use in genotyping other published *O. cumana* segregating populations and for diversity analyses, three further *O. cumana* populations used as parental lines in *O. cumana* crosses described in Rodríguez-Ojeda et al. (2013b) were used. These three populations belonged to the Guadalquivir Valley gene pool and were (i) OC-94 (race E, collected in Sevilla, Spain in 1994), (ii) EK-23 (race F, collected in Córdoba, Spain in 1995), and (iii) SP (race F, collected in Sevilla, Spain in 2004) (Rodríguez-Ojeda et al., 2013b).

### Tissue Collection and DNA Extraction

Broomrape shoots were first collected from individual F_2_ plants (single broomrape shoots), and individual plants from the parental lines EK-A1 and EK-12, and the other three *O. cumana* populations used for a polymorphism and diversity analysis (OC-94, EK-23, and SP). In order not to affect the F_3_ seed production, the apical bud of each F_2_ broomrape shoot was removed after most of the flowers had been formed. Because of the small amount of tissue collected in F_2_, tissue was also collected from F_2:3_ families (about 20 young F_3_ shoots per family) in order to recreate their corresponding F_2_ genotype. This is an accurate approach useful for species lacking enough tissue in individual F_2_ plants to yield enough DNA for marker analysis (Kochert, 1994). In this case, 20 young F_3_ shoots from each F_2:3_ family were collected before flowering, and bulked by mixing an equal tissue amount of the F_3_ shoots from each F_2:3_ family. In all cases, tissue was immediately frozen at -80°C, lyophilized, and ground in a laboratory ball mill. DNA was then extracted following the procedure reported by Pineda-Martos et al. (2013).

### Genotyping With SSR Markers

The complete set of 298 *O. cumana* SSR markers developed by Pineda-Martos et al. (2014) was tested for polymorphism in the parental lines EK-12 and EK-A1, and six individuals from the mapping population. PCR amplification was carried out in 30 μL reaction mixtures, consisting of 50 ng of template DNA, 0.03 U/μL of Taq DNA polymerase (FirePol Taq polymerase, Solis BioDyne, Tartu, Estonia), 1× PCR buffer, 2.5 mM MgCl_2_, 200 μM dNTPs (Solis BioDyne, Tartu, Estonia), and 0.3 μM of primers. A touchdown PCR program was used on a GeneAmp PCR System 9700 (Applied Biosystems, Foster City, CA, United States), which consisted of an initial denaturation step of 94°C for 2 min, followed by one cycle of 94°C for 30 s, final annealing temperature (*T_A_*) + 10°C for 30 s, and 72°C for 30 s, 9 cycles in which the annealing temperature was decreased by 1 °C, and 32 cycles at 94°C for 30 s, *T_A_* for 30 s, and 72°C for 30 s, with a final extension of 10 min at 72°C. Amplified products were separated on 3% MetaPhor (BMA, Rockland, ME, United States) agarose gels in 1× TBE buffer with SafeView Nucleic Acid Stain (NBS Biologicals Ltd., Huntingdon, United Kingdom) incorporated in the gel, in such a way that microsatellite alleles were effectively resolved with size differences between alleles by 2%. A 100 bp DNA ladder (Solis BioDyne, Tartu, Estonia) was used as a standard molecular weight marker to get an approximate size for the DNA fragments. The resultant gel images were scored manually with the aid of Quantity One 1-D Analysis Software (Bio-Rad Laboratories Inc., Hercules, CA, United States). The amplification profile for each microsatellite was scored visually and independently. A set of 28 polymorphic SSR markers showing clearly co-dominant and scorable fragments was selected and genotyped in the mapping population (91 individuals), following the same approach described above.

### Genotyping With SNP Markers

A set of 1536 *O. cumana* SNP markers developed by Coque et al. (2016) through exome capture was used to genotype the parental lines EK-12 and EK-A1 and the mapping population (ninety-one individuals) using competitive allele-specific PCR assays based on KASP^TM^ technology (LGC genomics, Teddington, United Kingdom) on the Limagrain genotyping platform. For this SNP discovery study, a set of 12 broomrape populations representing different level of virulence or aggressiveness and different countries was submitted to transcriptome sequencing. This approach led to the discovery of approximately 368000 bi-allelic SNP among which 1536 were selected for genotyping a Biogemma broomrape collection (around 500 populations). A genetic diversity analysis was conducted and an optimized subset of 198 SNP capturing the maximum of the genetic diversity analysis was selected. Details of the 198 *O. cumana* SNP markers are reported in Supplementary Table S1. The remainder of the SNP marker information is proprietary. For genetic mapping purpose, monomorphic markers were excluded for the linkage map construction.

In order to evaluate the potential of the SNPs reported in this study for their use in genotyping other *O. cumana* segregating populations, the number of markers polymorphic between parental genotypes of published mapping populations were determined as follows. The 198 *O. cumana* SNP marker subset from Supplementary Table S1 was genotyped in 10 individuals from each of the parental genotypes EK-A1, EK-12, EK-23, OC-94, and SP, used to develop the segregating populations EK-12 × EK-23, and OC-94 × SP, segregating for avirulence/virulence (Rodríguez-Ojeda et al., 2013b), and EK-A1 × EK-12, segregating for plant pigmentation (Rodríguez-Ojeda et al., 2011, this study). The individuals were genotyped for these SNP using the KASP^TM^ genotyping assay service provided by LGC Genomics (Herts, United Kingdom). A total of 38 markers and 7 individuals that failed genotyping or had >10% missing data, respectively, were excluded from the analysis. Therefore, polymorphism and genetic diversity analysis was carried out with 160 SNPs markers and the following parameters were calculated: percentage of polymorphic loci (P) and Shannon’s diversity index (I) within each population and percentage of polymorphic loci (P) between populations. In addition, pairwise genetic distances between populations were calculated as the genetic distance coefficient G_ST_ using 1000 random permutations to assess significance. The matrix of G_ST_ pairwise distances was used as input for a principal coordinates analysis (PCoA). All analyses were carried out using GenAlEx version 6.5 (Peakall and Smouse, 2012).

### Genetic Linkage Map Construction and Genetic Mapping of the Pigmentation Gene

Only co-dominant markers were used for the linkage map construction. The segregation of alleles at the SSR or SNP marker loci was checked against the expected ratios for codominant (1:2:1) markers using a chi-square test. The genetic linkage map was constructed with MAPMAKER/EXP (version 3.0b) (Lincoln et al., 1993) using genotyping data from polymorphic and co-dominant markers from the 298 SSR and 1536 SNP marker sets. Map distances in centiMorgan (cM) were converted from recombination fractions using the Kosambi mapping function. Two-point analysis was used to identify linkage groups (LGs) with an LOD score of 6.0 and a maximum distance of 40 cM, except for LGs 13 and 15, in which specific markers were grouped with a LOD value of 3.5. Three-point and multi-point analyses were used to determine the order and interval distances between the markers in each LG. Loci whose position were ambiguous (i.e., those placed automatically at a less-strict LOD of 2.0) were noted. Markers that had the most skewed segregation ratios (*P* < 0.0001) were excluded from the map. Linkage maps were drawn using MapChart 2.1 software (Voorrips, 2002). The linkage groups were randomly numbered as there are no previously reported *O. cumana* genetic maps. Simple correlation coefficients (r) between the total number of markers per linkage group and the total linkage group length were calculated. The significance of the correlation coefficients was calculated by the standard testing procedures for *r* = 0 null hypothesis (Snedecor and Cochran, 1989).

As the “plant pigmentation” trait is controlled by a single gene (*P_g_*, Rodríguez-Ojeda et al., 2011), it was mapped as a Mendelian locus. Accordingly, the genotypes for the *P_g_* gene were inferred from the pigmentation phenotypes in F_2_ plants and their corresponding F_2:3_ families. F_2_ plants were classified as homozygous dominant for the pigmentation gene if they showed a stem similar to EK-12, and showed uniformly pigmented plants in their respective F_3_ progeny, heterozygous if their F_3_ segregated for stem color, and homozygous recessive if they were similar to EK-A1 and showed uniformly unpigmented plants in their respective F_3_ progeny. Linkage analysis for the pigmentation gene was run with MAPMAKER/EXP (version 3.0b) using segregation data for SNP and SSR marker loci and for the *P_g_* locus. *P_g_* mapping was carried out as indicated for SSR and SNP markers, excepting that a LOD threshold of 10 and a maximum distance of 30 cM were used as linkage criteria. Finally, potential candidate genes for the *Pg* locus were identified using BLAST searches for plant pigment biosynthesis genes at the flavonoid/anthocyanin and carotenoid biosynthesis pathways (MetaCyc, version 20.0; Caspi et al., 2018) against a 622 contigs first draft of the *O. cumana* sequence genome (Gouzy et al., 2017).

## Results

### Phenotypic Evaluation of the Mapping Population

Phenotypic evaluation of the mapping population revealed 15 unpigmented F_2_ plants showing homogeneously unpigmented F_3_ progenies (a total of 675 unpigmented F_3_ plants for all the 15 F_2:3_ families) and 76 pigmented F_2_ plants. From these, 30 presented homogeneously bluish-violet pigmented F_3_ progenies (a total of 1146 bluish-violet F_3_ plants), and 46 showed segregating F_3_ progenies with both pigmented and unpigmented plants (a total of 1305 pigmented and 452 yellow-unpigmented F_3_ plants). The number of plants observed in each phenotypic class [15 (unpigmented): 46 (segregating): 30 (fully pigmented)] did not differ significantly from a 1:2:1 genetic proportion expected for one-gene segregation (χ^2^ = 4.96, *P* = 0.08).

### Genetic Linkage Map Construction

**FIGURE 2:**
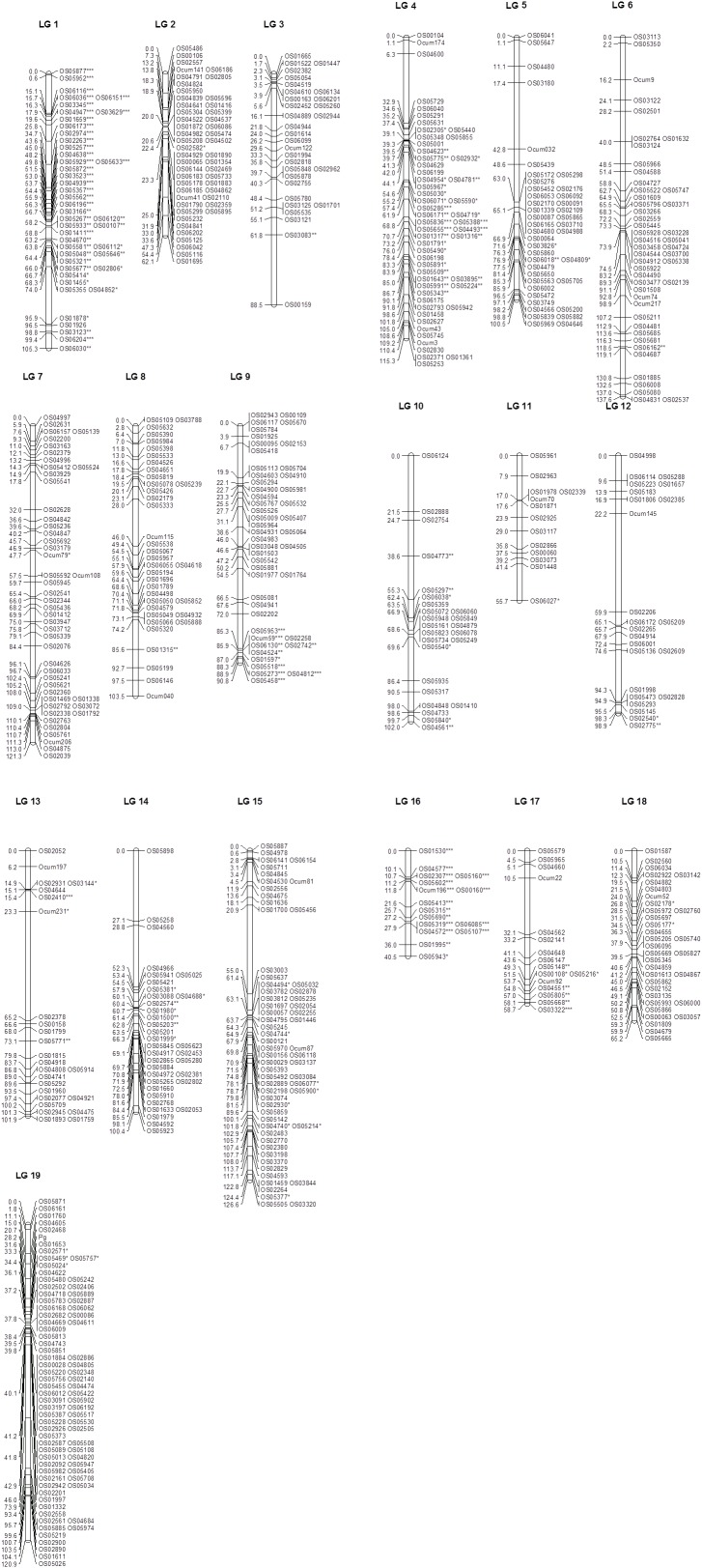
SSR and SNP *O. cumana* genetic linkage map containing the pigmentation locus *P_g_*. The map is based on segregation of 727 co-dominant SNP and SSR markers, and *P_g_*, in 91 individuals. Linkage groups (LG) follow a randomly selected numeration. Genetic distances are given in centiMorgans (Kosambi) on the left of each LG. The position of the *Pg* gene associated with plant pigmentation in *O. cumana*, mapped as a Mendelian trait, is shown at LG19. Prefix Ocum is for SSR marker loci and prefix OS for SNP marker loci. SNP and SSR marker loci labeled as ^∗^, ^∗∗^, or ^∗∗∗^ showed distorted segregation at *P* < 0.05, *P* < 0.01, and *P* < 0.001, respectively.

From the 1536 *O. cumana* SNPs, 1285 were successfully genotyped. Among these, 722 (56.2%) were polymorphic between the parents EK-A1 and EK-12 and segregated accordingly in the mapping population. From the 298 *O. cumana* SSRs, 168 showed high quality amplification, and 33 (19.6%) were polymorphic (28 co-dominant and 5 dominant). After excluding markers with extremely distorted segregation (*P* < 0.0001) and dominant markers, 737 markers were used for the linkage analysis. The final genetic linkage map was constructed using 26 SSR and 701 SNP polymorphic and codominant markers. Two additional markers that remained unlinked and 8 markers that remained “unmapped” (grouped in LGs of two or three markers) were not included. The 727 SSR and SNP loci were arranged in 19 linkage groups, which correspond to the 19 chromosome pairs of the *O. cumana* genome (Schneeweiss et al., 2004; Piednoël et al., 2012; Figure 2 and Supplementary Table S2). The total map length was 1795.7 cM. The average distance between two adjacent positions across the whole map was 2.5 cM, but there were 12 regions on 11 LGs with intervals greater than 20 cM, with the largest interval (41.9 cM) being observed in LG13 (Figure 2 and Supplementary Table S2).

The number of markers on different LGs ranged from 13 on LG11 to 80 on LG19. The length of different LGs ranged from 40.5 cM on LG16 to 137.6 cM on LG6 (Figure 2 and Supplementary Table S2). The LG length was correlated with the number of markers per linkage group (*r* = 0.66, *p* = 0.002, *n* = 19). LG2 was the most skewed linkage group from the linear fitting, probably due to an excess of markers (a total of 28) clustering in two groups of 0 cM (Figure 2). Excluding this LG resulted in a higher correlation (*r* = 0.74, *p* = 0.0004, *n* = 18). A highly significant (*P* < 0.001) distorted segregation was observed in LG1, LG4, the bottom of LG9, and LG16 (Supplementary Table S2).

### Genetic Mapping of the P_g_ Pigmentation Gene

Using the genotypic classification of F_2_ plants (homozygous unpigmented: segregating: homozygous pigmented) based on the F_3_ evaluation, the locus *P_g_* associated with plant pigmentation mapped as a Mendelian trait was located 28.2 cM downstream from the upper end of LG19, between the SNP markers OS02468 and OS01653, which were 7.5 cM distal and 3.4 cM proximal, respectively, of the *P_g_* locus (Figure 2). Based on the physical position of context sequences of markers flanking *Pg* on the *O. cumana* draft genome (OS01653 and OS02571 at positions 4.17 and 4.07 Mbp, respectively, in contig OcIN23s039, and OS02468 and OS04605 at positions 2.51 and 4.41 Mbp, respectively, in contig OcIN23s036), candidate genes for plant pigment biosynthesis at the flavonoid/anthocyanin and carotenoid biosynthesis pathways were searched locating at these contigs. Two contiguous genes for flavonoid biosynthesis and carotenoid biosynthesis were found in contig OcIN23s036. Both genes were coding for carotenoid/flavonoid glucoside glucosyltransferases. The genes were a flavonoid glucoside glucosyltransferase (at position 3.61 Mbp) and a crocetin glucoside glucosyltransferase (at position 3.60 Mbp) whose physical position was between that of OS02468 and OS04605 SNP markers in contig OcIN23s036.

### Diversity and Polymorphism SNP Analysis

**FIGURE 3:**
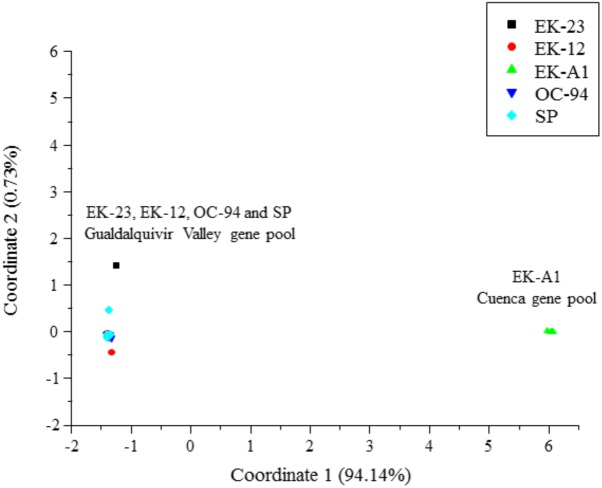
Principal coordinates analysis of the five *O. cumana* parental genotypes EK-A1, EK-12, EK-23, OC-94, and SP.

Evaluation of the potential of SNPs (using the 198 SNP subset from Supplementary Table S1) for genotyping existing segregating populations in *O. cumana* revealed an extremely low number of polymorphic markers between parental genotypes coming from populations of the Guadalquivir Valley gene pool (OC-94, SP, EK-12, and EK-23), in contrast to a much higher number of polymorphic markers found between EK-A1 from the Cuenca gene pool and the remaining genotypes from the Guadalquivir Valley gene pool, including EK-12 (Table 1). This was coupled with a very low intra-population variation and inter-population diversity for populations from the same gene pool, as shown by diversity parameters (Table 1). Principal Coordinate Analysis revealed a clear separation between parental genotypes from the Guadalquivir Valley gene pool (OC-94, SP, EK-12, and EK-23), and the Cuenca gene pool (EK-A1) (Figure 3), separated along Coordinate 1 that explained 94.14% of the total variation.

**Table 1:** Percentage of polymorphic SNP loci between the parental genotypes EK-A1 (Cuenca gene pool), EK-12 (Guadalquivir valley gene pool), EK-23 (Guadalquivir valley gene pool), OC-94 (Guadalquivir valley gene pool), and SP (Guadalquivir valley gene pool) from *O. cumana* segregating populations.

	EK-A1	EK-12	EK-23	OC-94	SP
EK-A1	0.00%; 0.000	56.88%	56.88%	56.88%	56.88%
EK-12		0.63%; 0.004	0.63%	1.88%	0.63%
EK-23			0.63%; 0.004	1.88%	0.63%
OC-94				1.88%; 0.008	1.88%
SP					0.63%; 0.004

*In the diagonal axis intra-population genetic diversity parameters are indicated as follows: Percentage of polymorphic loci and Shannon’s diversity index.*

## Discussion

Genetic studies in *O. cumana* have been very scarce till date. Rodríguez-Ojeda et al. (2011) studied the inheritance of the unpigmented plant trait in line EK-A1. The trait was found to be controlled by partially dominant alleles at a single locus, named *P_g_*. Another genetic study at the phenotypic level was conducted by Rodríguez-Ojeda et al. (2013b), who evaluated the segregation of virulence in progenies from crosses between race E and race F lines developed from populations collected in southern Spain. Differences in virulence between both types of populations were found to be controlled by a single gene, suggesting that the evolution from race E to race F in that area was produced by a point mutation. Genetic studies in *O. cumana*, conducted initially at the phenotypic level, can be now be expanded at the molecular level thanks to genetic and genomic advances in this species, such as the development of SSR markers (Pineda-Martos et al., 2014), SNP markers (reported for the first time in this study), or a first draft of the genome sequence (Gouzy et al., 2017). The present study, in which SNP markers have been reported, a genetic linkage map using SSR and SNPs has been constructed and the *Pg* gene (characterized previously at the phenotypic level by Rodríguez-Ojeda et al., 2011) mapped, is in the same line as these previous studies, providing new genetic tools for understanding genetic mechanisms in this parasitic weed, and ultimately contributing to the development of durable genetic resistance in sunflower.

The development of a genetic linkage map is an important pre-requisite for the identification of genes or QTL underlying important traits. The *O. cumana* map developed in the present research is the first linkage map reported for any *Orobanche/Phelipanche* species and represents a significant advance in the study of genome structure and organization, and for mapping genes of importance on the *O. cumana* genome. This *O. cumana* genetic map contains 19 LGs, coincident with the basic number of chromosomes in this species (Schneeweiss et al., 2004; Piednoël et al., 2012), a relatively high marker density (a total of 727 SNP and SSR marker loci) and a resolution of 2.5 cM. From 737 polymorphic markers used for linkage analysis, only 10 remained “unmapped,” with 98.6% of the markers falling into one larger LG (with a minimum number of markers of 13, and coverage >40 cM). Therefore, the map developed appears to cover the vast majority of the *O. cumana* genome. The genetic linkage map information from this study combined with the availability of an *O. cumana* genome sequence draft and the contig and genetic linkage position of SNP and SSR markers, will be of utility to anchor contigs into scaffolds and ultimately to obtain the chromosome sequences, contributing in this way to the development of new genetic resources for this species.

The total *O. cumana* map length was 1795.7 cM. Considering the size of its genome to be 1.42 Gb or 1.40 Gb resulting from either densitometry (Weiss-Schneeweiss et al., 2006) or assembly of DNA sequences (Pouilly et al., 2018), respectively, the *O. cumana* genome-wide recombination rate was estimated as 1.27 cM/Mb. This value is slightly lower than the average value of 1.85 cM/Mb previously reported by Stapley et al. (2017) for plants (averaged from 189 species), and also lower than the average value of 2.6 cM/Mb (ranging from 1.6 to 3.9 cM/Mb) detailed by these authors for five plant species of the Lamiales order, to which the *Orobanchaceae* family belongs. Interestingly, Stapley et al. (2017) compared the genome-wide recombination rate of parasitic or pathogenic species with free-living species and found that parasitic or pathogenic species had a higher recombination rate compared to their free-living counterparts in animals, but they found no differences in fungi. Unfortunately, no data were reported for parasitic plants as parasitic/pathogenic plant species were not included in their dataset.

The distribution of markers between the 19 linkage groups was fairly uniform with, in general, the largest groups containing the most markers. However, there were markers clustered observed in some regions of the map, especially on LG2, LG5, and LG19. Marker clustering has also been reported in genetic maps in other plant species, and it has been associated with centromeric regions due to the suppression of recombination in the heterochromatic regions surrounding these regions (Haanstra et al., 1999), or to the lack of recombination around genes with evolutionary significance (Jessup et al., 2002; Hao et al., 2004). In addition, despite the short average distance between adjacent markers on the map (2.5 cM), there were 12 gaps larger than 20 cM. These gaps have also been reported in several genetic linkage maps in plant species and may be due to the lack of marker polymorphism and a shortage of marker detection in these regions (Berry et al., 1995) or due to recombination hot spots (Mézard, 2006).

Around 20.5% (149 out of 728) of the mapped markers in the map had significantly distorted segregation ratios. They were mainly clustered on LG1 and LG16 and on specific regions in LG4 and LG9, suggesting that these may be of biological significance. Distorted segregation in specific genomic regions is an inevitable feature observed in many of the marker-based linkage maps in plants. It has been attributed to a range of causes, including deleterious recessive alleles (Berry et al., 1995), self-incompatibility alleles (Barzen et al., 1995), structural rearrangements (Quillet et al., 1995), or differences in DNA content (Jenczewski et al., 1997). It is worth noting that the allele differences that contribute to the relative success of parasitism could also lead to segregation distortion (Thomas et al., 2012). Interestingly, all markers showing segregation distortion clustering at LG1, LG9, and LG16 favored the allele from the pigmented parent EK-12. Although the pigmentation gene *P_g_* did not map in any of those regions, and the lack of pigmentation in *O. cumana* plants has not been associated to parasitism (González-Torres et al., 1982; Rodríguez-Ojeda et al., 2011), this might suggest a fitness benefit for progenies that inherited specific EK-12 alleles.

Overall SNP polymorphism in the EK-A1 and EK-12 cross was relatively high (more than 50% polymorphic loci). This cross involved parents from two distant gene pools identified in Spain, with the EK-A1 population belonging to the Cuenca gene pool in Central Spain and the EK-12 population being from the Guadalquivir Valley gene pool in Southern Spain (Rodríguez-Ojeda et al., 2011; Pineda-Martos et al., 2013). In addition to these two divergent gene pools in Spain, Pineda-Martos et al. (2013) also described an extremely low inter- and intra- population diversity within each of these two gene pools using SSR markers, which was attributed to a founder effect. This lack of polymorphism within the Guadalquivir Valley gene pool has also been confirmed in this study using SNP markers. Therefore, using parental lines from already described *O. cumana* distant gene pools to avoid factors decreasing diversity such as founder effects is desirable for genetic mapping and construction of highly saturated genetic maps in *O. cumana*, since the possibility of detecting polymorphism among parents is increased, resulting in a higher number of segregating loci.

*Orobanche* spp. are often highly variable in regard to their size, coloration, and pubescence (Rumsey and Jury, 1991). Unpigmented plants (likely lacking anthocyanin pigmentation) have been observed in the populations of several *Orobanche* spp. (Kreutz, 1995; Rumsey, 2007), including *O. cumana* (González-Torres et al., 1982). Results previously reported by Rodríguez-Ojeda et al. (2011) showed that the absence of pigmentation in *O. cumana* was the result of a single-gene mutation, probably involved in anthocyanin biosynthesis. Later, Rodríguez-Ojeda et al. (2013a) proved the unpigmented trait in *O. cumana* to be very useful for studies on its biology, allowing the determination of cross-fertilization rate in this species. In this study, the pigmentation locus *P_g_* has been mapped to LG19 in the SSR-SNP *O. cumana* genetic linkage map, between the SNP markers OS01653 and OS02468. This constitutes the first trait mapping study in *Orobanche* spp. Even though the development of molecular markers for the pigmentation trait may not have drawn interest because the trait is easily distinguishable visually, the location of a gene with a known phenotypic effect in the *O. cumana* genetic map may be an important reference for future mapping and molecular marker studies in this species. In addition, it might contribute to the identification of causal genes and mutations for stem and flower-color variations in *O. cumana*. In fact, candidate gene analysis revealed two contiguous genes for carotenoid/flavonoid glucoside glucosyltransferases mapping close to the *Pg* locus. Glycosylation is often the final step in the biosynthesis of plant secondary metabolites, which enhances their water solubility and chemical stability and alters their biological activity (Yang et al., 2018). The attachment of additional sugar to flavonoid glycosides has also been related to modifications in physiological properties such as color. For instance, anthocyanin color is influenced by glycosylation pattern (Zhang et al., 2014), and its possible role on *Orobanche* plant pigmentation might be further investigated.

## Conclusion

In conclusion, this work represents the first genetic linkage map and trait mapping study for *O. cumana*, and for any *Orobanche/Phelipanche* spp., a species and a genus for which there are very limited genetic/genomic information published. Results from this study will contribute to understand the genetic basis of the sunflower- *O cumana* interaction, which is required for the development of new knowledge-based strategies for broomrape management. In this sense, the reported SNPs and the saturated genetic map constitute valuable genetic resources for different downstream applications such as new SNP-based genetic diversity and population structure analyses, further genome characterization and sequence assembly of the *O. cumana* genome, and the identification of genes/QTL underlying relevant traits. The mapping of the *P_g_* locus determining plant pigmentation provides one example, and mapping genes/QTL associated to parasitism and virulence using segregating populations generated from parental lines differing for these traits is under way. Locating and eventually cloning genes responsible for these traits will bear direct implications for practical agriculture, since they will represent new targets for rational design of control strategies to this devastating parasitic weed.

## Author Contributions

LV and BP-V conceived the study and planned and supervised the research. SM supervised a part of the genotyping. MC conducted the SNP development and genotyping. ÁC-G and NP conducted the SSR genotyping and collaborated in the SNP genotyping. BP-V, XG, and ÁC-G conducted the statistical analyses and map construction. ÁC-G, LV, and BP-V wrote the draft of the manuscript. All authors critically read the manuscript and revised its final version.

## Conflict of Interest Statement

The authors declare that the research was conducted in the absence of any commercial or financial relationships that could be construed as a potential conflict of interest.

## Supplementary Material

TABLE S1 Details of 198 *O. cumana* SNP markers, including their context sequence and map positions.

TABLE S2 Map positions of 727 SNP and SSR marker loci and the pigmentation locus Pg in the *O. cumana* genetic linkage map. Prefix Ocum is for SSR marker loci and prefix OS for SNP marker loci. Marker loci labelled as ^∗^, ^∗∗^, or ^∗∗∗^ showed distorted segregation at *P* < 0.05, *P* < 0.01, or *P* < 0.001, respectively. Loci whose position were ambiguous (i.e., those placed automatically at a less-strict LOD of 2.0) were labelled in red. Markers highlighted in bold and underlined are SSR or those SNP detailed in Supplementary Table S1.

## References

[B1] Amri M. Abbes Z. Ben Youssef S. Bouhadida M. Ben Salah H. Kharrat M. (2012). Detection of the parasitic plant, *Orobanche cumana* on sunflower (*Helianthus annuus* L.) in Tunisia. *Afr. J. Biotechnol.* 11 4163–4167. 10.5897/AJB11.3031

[B2] Antonova T. S. Araslanova N. M. Strelnikov E. A. Ramazanova S. A. Guchetl S. Z. Chelyustnikova T. A. (2013). Distribution of highly virulent races of sunflower broomrape (*Orobanche cumana* Wallr.) in the Southern regions of the Russian Federation. *Russ. Agric. Sci.* 39 46–50. 10.3103/S1068367413010023

[B3] Barzen E. Mechelke W. Ritter E. Schulte-Kappert E. Salamini F. (1995). An extended map of the sugar beet genome containing AFLP and RFLP loci. *Theor. Appl. Genet.* 90 189–193. 10.1111/j.1439-0523.1993.tb00641.x 24173890

[B4] Berry S. T. Leon A. J. Hanfrey C. C. Challis P. Burkholz A. Barnes S. R. (1995). Molecular marker analysis of *Helianthus annuus* L. 2. Construction of an RFLP linkage map for cultivated sunflower. *Theor. Appl. Genet.* 91 195–199. 10.1007/BF00220877 24169763

[B5] Caspi R. Billington R. Fulcher C. A. Keseler I. M. Kothari A. Krummenacker M. (2018). The MetaCyc database of metabolic pathways and enzymes. *Nucleic Acids Res.* 46 D633–D639. 10.1093/nar/gkx935 29059334 PMC5753197

[B6] Castejón-Muñoz M. Suso M. J. Romero-Muñoz F. García-Torres L. (1991). “Isoenzymatic study of broomrape (*Orobanche cernua*) populations infesting sunflower (*Helianthus annuus*),” in *Proceedings of the 5th International Symposium on Parasitic Weeds*, eds Ransom J. K. Musselman L. J. Worsham A. D. Parker C. (Nairobi: International Maize and Wheat Improvement Center – CIMMYT), 313–319.

[B7] Coque M. André T. Giménez R. Archipiano M. Polovynko L. Tardin M. C. (2016). “Study of Orobanche cumana genetic diversity,” in *Proceedings of the 19th International Sunflower Conference*, eds Kaya Y. Hasancebi S. (Paris: International Sunflower Association), 734.

[B8] Fernández-Escobar J. Rodríguez-Ojeda M. I. Fernández-Martínez J. M. Alonso L. C. (2009). Sunflower broomrape (*Orobanche cumana* Wallr.) in Castilla-León, a traditionally non-broomrape infested area in Northern Spain. *Helia* 51 57–64. 10.2298/HEL0951057F

[B9] Fernández-Martínez J. M. Pérez-Vich B. Velasco L. (2015). “Sunflower broomrape (*Orobanche cumana* Wallr.),” in *Sunflower Oilseed. Chemistry, Production, Processing and Utilization*, eds Martínez-Force E. Dunford N. T. Salas J. J. (Champaign, IL: AOCS Press), 129–156.

[B10] Gagne G. Roeckel-Drevet P. Grezes-Besset B. Shindrova P. Ivanov P. Grand-Ravel C. (1998). Study of the variability and evolution of *Orobanche cumana* populations infesting sunflower in different European countries. *Theor. Appl. Genet.* 96 1216–1222. 10.1007/s001220050859

[B11] González-Torres R. Jiménez-Díaz R. M. Melero-Vara J. M. (1982). Distribution and virulence of *Orobanche cernua* in sunflower crops in Spain. *Phytopathologische Zeitschrift* 104 78–89. 10.1111/j.1439-0434.1982.tb00008.x

[B12] Gouzy J. Pouilly N. Boniface M. C. Bouchez O. Carrère S. Catrice O. (2017). “The complete genome sequence of *Orobanche cumana* (sunflower broomrape),” in *Proceedings of the 14th World Congress on Parasitic Plants*, (Sheffield: International Plant Parasitic Society).

[B13] Guchetl S. Z. Antonova T. S. Tchelustnikova T. A. (2014). Genetic similarity and differences between the *Orobanche cumana* wallr. populations from Russia, Kazakhstan, and Romania revealed using the markers of simple sequence repeat. *Russ. Agric. Sci.* 40 326–330. 10.3103/S1068367414050103

[B14] Haanstra J. P. W. Wye C. Verbakel H. Meijer-Dekens F. Berg P. V. D. Odinot P. (1999). An integrated high-density RFLP-AFLP map of tomato based on two *lycopersicon esculentum* ×*L. pennellii* F2 populations. *Theor. Appl. Genet.* 99 254–271. 10.1007/s001220051231

[B15] Hao M. Moore P. H. Liu Z. Kim M. S. Yu Q. Fitch M. M. M. (2004). High-density linkage mapping revealed suppression of recombination at the sex determination locus in papaya. *Genetics* 166 419–436. 10.1534/genetics.166.1.419 15020433 PMC1470706

[B16] Jenczewski E. Gherardi M. Bonnin I. Prosperi J. M. Olivieri I. Huguet T. (1997). Insight on segregation distortions in two intraspecific crosses between annual species of *Medicago*(Leguminosae). *Theor. Appl. Genet.* 94 682–691.

[B17] Jessup R. W. Burson B. L. Burow G. B. Wang Y.-W. Chang C. Li Z. (2002). Disomic inheritance, suppressed recombination, and allelic interaction govern apospory in buffelgrass as revealed by genome mapping. *Crop Sci.* 42 1688–1694. 10.2135/cropsci2002.1688

[B18] Jestin C. Lecomte V. Duroueix F. (2014). “Current situation of sunflower broomrape in France,” in *Proceedings of the 3rd International Symposium on Broomrape (Orobanche spp.) in Sunflower*, (Paris: International Sunflower Association), 28–31.

[B19] Kaya Y. Evci G. Pekcan V. Gucer T. Yilmaz M. I. (2009). Evaluation of broomrape resistance in sunflower hybrids. *Helia* 51 161–170. 10.2298/hel0951161k

[B20] Kochert G. (1994). “RFLP technology,” in *DNA-Based Markers in Plants*, eds Phillips R. L. Vasil K. (Dordrecht: Springer), 8–38.

[B21] Kreutz C. A. J. (1995). *Orobanche: The European Broomrape Species. I. Central and Northern Europe.* Maastricht: Stichting Natuurpublicaties Limburg.

[B22] Lincoln S. Daley M. Lander E. (1993). *Constructing Genetic Linkage Map with Mapmaker/exp. 3.0: A Tutorial and Reference Manual*, 3rd Edn. Cambridge: Whitehead Institute for Biometrical Research.

[B23] Malek J. del Moral L. Fernández-Escobar J. Pérez-Vich B. Velasco L. (2017). Racial characterization and genetic diversity of sunflower broomrape populations from Northern Spain. *Phytopathol. Mediterr.* 56 70–76. 10.14601/Phytopathol_Mediterr-19163

[B24] Martín-Sanz A. Malek J. Fernández-Martínez J. M. Pérez-Vich B. Velasco L. (2016). Increased virulence in sunflower broomrape (*Orobanche cumana* Wallr.) populations from Southern Spain is associated with greater genetic diversity. *Front. Plant Sci.* 7:589 10.3389/fpls.2016.00589 PMC4853410 27200060

[B25] Mézard C. (2006). Meiotic recombination hotspots in plants. *Biochem. Soc. Trans.* 34 531–534. 10.1042/BST0340531 16856852

[B26] Molinero-Ruiz M. L. García-Carneros A. B. Collado-Romero M. Raranciuc S. Domínguez J. Melero-Vara J. M. (2014). Pathogenic and molecular diversity in highly virulent populations of the parasitic weed *Orobanche cumana* from Europe. *Weed Res.* 54 87–96. 10.1111/wre.12056

[B27] Nabloussi A. Velasco L. Assissel N. (2018). First report of sunflower broomrape, *Orobanche cumana* Wallr., in Morocco. *Plant Dis.* 102:457 10.1094/PDIS-06-17-0858-PDN

[B28] Pacureanu-Joita M. Raranciuc S. Sava E. Stanciu D. Nastase D. (2009). Virulence and aggressiveness of sunflower broomrape (*Orobanche cumana* Wallr.) populations in Romania. *Helia* 51 111–118. 10.2298/hel0951111p

[B29] Pacureanu-Joita M. Veronesi C. Raranciuc S. Stanciu D. (2004). “Parasite-host plant interaction of Orobanche cumana Wallr. (Orobanche cernua Loefl) with Helianthus annuus,” in *Proceedings of the 16th International Sunflower Conference*, ed. Seiler G. J. (Paris: International Sunflower Association),171–177.

[B30] Peakall R. Smouse P. E. (2012). GenAlEx 6.5: genetic analysis in Excel. Population genetic software for teaching and research – an update. *Bioinformatics* 28 2537–2539. 10.1093/bioinformatics/bts460 22820204 PMC3463245

[B31] Pescott O. (2013). *The Genetics of Host Adaptation in the Parasitic Plant Striga Hermonthica.* Ph.D. thesis, The University of Sheffield, Sheffield.

[B32] Piednoël M. Aberer A. J. Schneeweiss G. M. Macas J. Novák P. Gundlach H. (2012). Next-generation sequencing reveals the impact of repetitive DNA across phylogenetically closely related genomes of Orobanchaceae. *Mol. Biol. Evol.* 29 3601–3611. 10.1093/molbev/mss168 22723303 PMC3859920

[B33] Pineda-Martos R. Velasco L. Fernández-Escobar J. Fernández-Martínez J. M. Pérez-Vich B. (2013). Genetic diversity of sunflower broomrape (*Orobanche cumana*) populations from Spain. *Weed Res.* 53 279–289. 10.1111/wre.12022

[B34] Pineda-Martos R. Velasco L. Pérez-Vich B. (2014). Identification, characterization, and discriminatory power of microsatellite markers in the parasitic weed *Orobanche cumana*. *Weed Res.* 54 120–132. 10.1111/wre.12062

[B35] Pouilly N. Gouzy J. Boniface M. C. Bouchez O. Carrère S. Catrice O. (2018). “The complete genome sequence of the parasitic weed Orobanche cumana (sunflower broomrape),” in *Proceeding of the XXVI Plant and Animal Genome Conference XXVI*, (San Diego),

[B36] Quillet M. C. Madjidian N. Griveau Y. Serieys H. Tersac M. Lorieux M. (1995). Mapping genetic factors controlling pollen viability in an interspecific cross in *Helianthus* sect. *Helianthus*. *Theor. Appl. Genet.* 91 1195–1202. 10.1007/BF00220929 24170046

[B37] Rodríguez-Ojeda M. I. Pérez-Vich B. Alonso L. C. Fernández-Escobar J. (2010). The influence of flowering plant isolation on seed production and seed quality in *Orobanche Cumana*. *Weed Res.* 50 145–150. 10.1111/j.1365-3180.2010.00817.x

[B38] Rodríguez-Ojeda M. I. Fernández-Martínez J. M. Velasco L. Pérez-Vich B. (2013a). Extent of cross-fertilization in *Orobanche cumana* Wallr. *Biol. Plantarum* 57 559–562. 10.1007/s10535-012-0301-1

[B39] Rodríguez-Ojeda M. I. Pineda-Martos R. Alonso L. C. Fernández-Escobar J. Fernández-Martínez J. M. Pérez-Vich B. (2013b). A dominant avirulence gene in *Orobanche cumana* triggers *Or5* resistance in sunflower. *Weed Res.* 53 322–327. 10.1111/wre.12034

[B40] Rodríguez-Ojeda M. I. Velasco L. Alonso L. C. Fernández-Escobar J. Pérez-Vich B. (2011). Inheritance of the unpigmented plant trait in *Orobanche cumana*. *Weed Res.* 51 151–156. 10.1111/j.1365-3180.2010.00830.x

[B41] Rumsey F. J. (2007). A reconsideration of *Orobanche maritima* Pugsley (Orobanchaceae) and related taxa in southern England and the Channel Islands. *Watsonia* 26 473–476.

[B42] Rumsey F. J. Jury S. L. (1991). An account of *Orobanche* L. in Britain and Ireland. *Watsonia* 18 257–295.

[B43] Schneeweiss G. M. Palomeque T. Colwell A. E. Weiss-Schneeweiss H. (2004). Chromosome numbers and karyotype evolution in holoparasitic *Orobanche* (Orobanchaceae) and related genera. *Am. J. Bot.* 91 439–448. 10.3732/ajb.91.3.439 21653400

[B44] Shindrova P. Penchev E. (2012). Race composition and distribution of broomrape (*Orobanche cumana* Wallr.) in Bulgaria during 2007-2011. *Helia* 57 87–94. 10.2298/hel1257087s

[B45] Snedecor G. W. Cochran W. G. (1989). *Statistical Methods*, 8th Edn. Ames, IA: Iowa State University Press.

[B46] Stapley J. Feulner P. G. D. Johnston S. E. Santure A. W. Smadja C. M. (2017). Variation in recombination frequency and distribution across eukaryotes: patterns and processes. *Philos. Trans. R. Soc. B* 372:20160455 10.1098/rstb.2016.0455 PMC5698618 29109219

[B47] Thomas V. P. Fudali S. L. Schaff J. E. Liu Q. Scholl E. H. Opperman C. H. (2012). A sequence-anchored linkage map of the plant-parasitic nematode *Meloidogyne hapla* reveals exceptionally high genome-wide recombination. *Genes Genomes Genet.* 2 815–824. 10.1534/g3.112.002261 PMC3385987 22870404

[B48] Velasco L. Pérez-Vich B. Yassein A. A. M. Jan C. C. Fernández-Martínez J. M. (2012). Inheritance of resistance to sunflower broomrape (*Orobanche cumana* Wallr.) in a interspecific cross between *Helianthus annuus* and *Helianthus debilis* subsp. *tardiflorus*. *Plant Breed.* 121 220–221. 10.1111/j.1439-0523.2011.01915.x

[B49] Voorrips R. E. (2002). MapChart: software for the graphical presentation of linkage maps and QTLs. *J. Hered.* 93 77–78. 10.1093/jhered/93.1.77 12011185

[B50] Vranceanu A. V. Tudor V. A. Stoenescu F. M. Pirvu N. (1980). “Virulence groups of Orobanche cumana Wallr. differential hosts and resistance sources and genes in sunflower,” in *Proceedings of the 9th International Sunflower Conference*, (Paris: International Sunflower Association), 74–80.

[B51] Weiss-Schneeweiss H. Greilhuber J. Schneeweiss G. M. (2006). Genome size evolution in holoparasitic *Orobanche* (Orobanchaceae) and related genera. *Am. J. Bot.* 93 148–156. 10.3732/ajb.93.1.148 21653400

[B52] Yang B. Liu H. Yang J. Gupta V. K. Jiang Y. (2018). New insights on bioactivities and biosynthesis of flavonoid glycosides. *Trends Food Sci. Technol.* 79 116–124. 10.1016/j.tifs.2018.07.006

[B53] Zhang Y. Butelli E. Martin C. (2014). Engineering anthocyanin biosynthesis in plants. *Curr. Opin. Plant Biol.* 19 81–90. 10.1016/j.pbi.2014.05.011 24907528

